# Post-transcriptional regulation by HuR in colorectal cancer: impacts on tumor progression and therapeutic strategies

**DOI:** 10.3389/fimmu.2025.1658526

**Published:** 2025-10-22

**Authors:** Yilin Shi, Zhen Zhou, Cong Liu, Jing Liu, Mengying Xie, Xin Chen, Dan A. Dixon, Xiaoqing Wu, Lingling Yang

**Affiliations:** ^1^ Department of Gastroenterology, The Second Affiliated Hospital, Jiangxi Medical College, Nanchang University, Nanchang, Jiangxi, China; ^2^ Division of Gastrointestinal Surgery Center, The First Affiliated Hospital of Sun Yat-sen University, Guangzhou, Guangdong, China; ^3^ Department of Pathology Surgery, The Second Affiliated Hospital, Jiangxi Medical College, Nanchang University, Nanchang, Jiangxi, China; ^4^ Institute of Pharmaceutical Biotechnology, School of Basic Medical Sciences, Zhejiang University, Hangzhou, Zhejiang, China; ^5^ Department of Biochemistry and Molecular Biology, University of Arkansas for Medical Sciences, Little Rock, AR, United States; ^6^ Department of Molecular Biosciences, The University of Kansas, Lawrence, KS, United States

**Keywords:** colorectal cancer, RNA-binding proteins, human antigen R, post-transcriptional regulation, therapeutic strategies

## Abstract

Colorectal cancer (CRC) is the third most common malignancy worldwide and the second leading cause of cancer-related deaths. Its progression is driven by genetic and epigenetic alterations, with increasing evidence emphasizing the role of the transcriptome, particularly post-transcriptional modifications. Human antigen R (HuR), an RNA-binding protein (RBP), plays a crucial role in post-transcriptional regulation of gene expression. In the context of tumor progression, HuR affects a range of cellular processes, including cell proliferation, survival, and metabolic reprogramming, via regulating target mRNA stability and translation. Additionally, HuR influences the tumor microenvironment (TME) through modulating target mRNAs involved in inflammation, immune responses, extracellular matrix remodeling and angiogenesis. Despite these insights, the precise mechanisms by which HuR regulates post-transcriptional process in CRC remain unclear. This review first provides an overview of HuR’s roles and the underlying mechanisms involved in CRC progression, including its regulation of mRNA expression, control of the cell cycle, and modulation of the TME. We also discussed the potential of HuR as a therapeutic target, exploring how targeting HuR could slow down CRC progression and metastasis, ultimately leading to more effective and personalized treatment strategies.

## 1 Introduction

Colorectal cancer (CRC) is the third most common malignancy worldwide and remains a significant cause of cancer-related mortality (1). Despite advancements in CRC diagnosis and surgical treatment, its incidence and mortality rates have only slightly declined over the past two decades. A major challenge in CRC management is the difficulty of early detection, as its symptoms are often nonspecific (2). Moreover, metastasis, recurrence, and drug resistance remain significant hurdles in achieving effective treatment (3). Consequently, a deeper understanding of the mechanisms driving CRC development and progression is critical for improving therapeutic strategies and patient prognoses.

Recent advancements in molecular biology and genomics have enhanced our understanding of CRC pathogenesis, particularly in gene expression regulation (4). RNA-binding proteins (RBPs), essential post-transcriptional regulators, play a pivotal role in regulating RNA localization, stability, and translation (5). These RBPs affect the expression of a wide array of mRNA transcripts and long non-coding RNAs (lncRNAs), thereby influencing various cellular processes in cancer progression (6–8). In CRC, RBPs serve as critical epigenetic regulators that can modify the expression of target genes, subsequently affecting tumor growth, metastasis, and patient prognosis (9, 10). Moreover, recent studies indicate that RBPs also influence key components of the tumor microenvironment (TME), further promoting CRC progression (11, 12).

**Figure 1:**
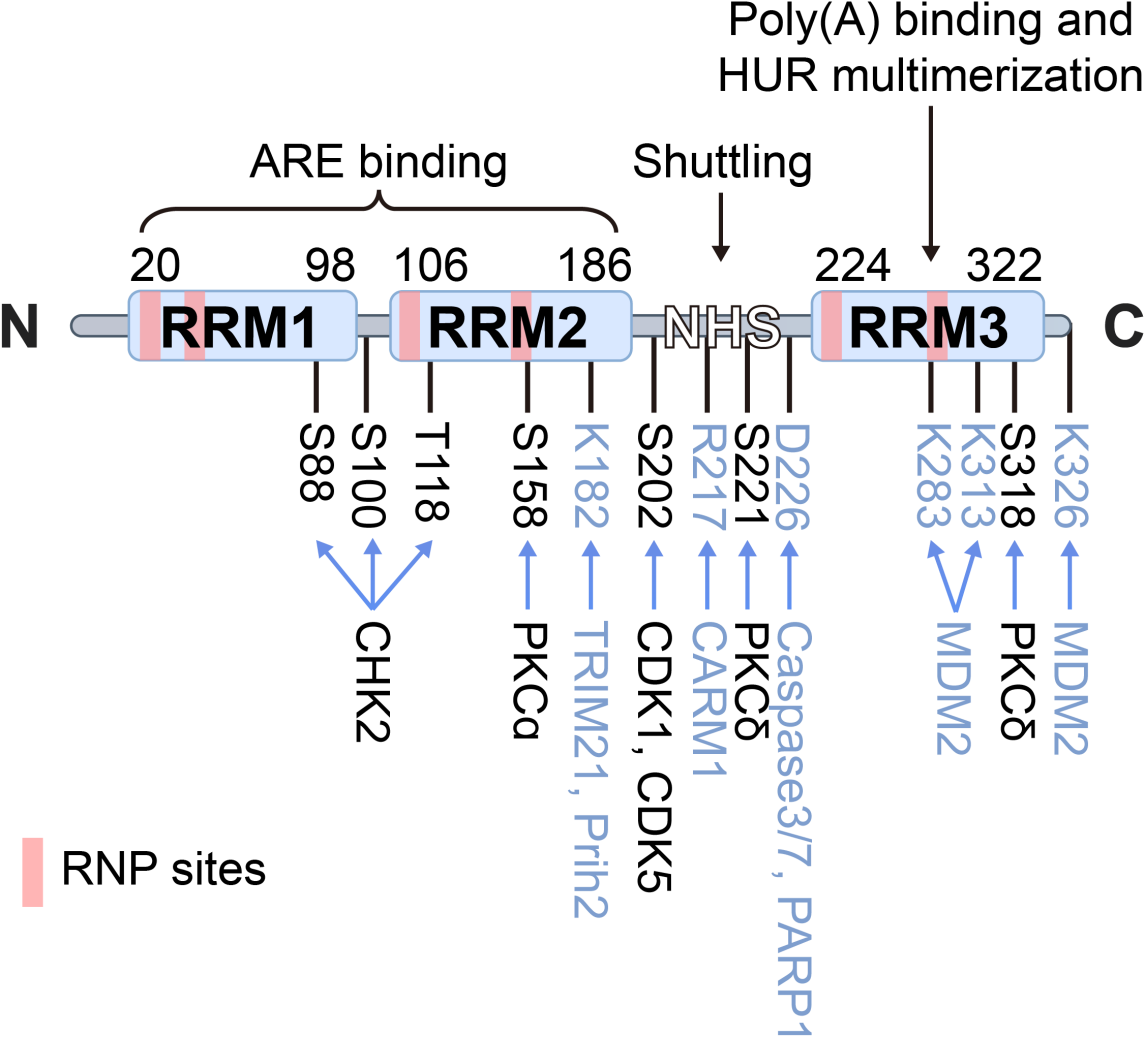
Schematic structure of HuR. HuR is a 326-aa protein containing three RNA recognition motifs (RRM1–3) and a hinge region (HNS) with a nucleocytoplasmic shuttling sequence. RRM1/2 bind AU-rich elements (AREs) in target mRNAs, while RRM3 contributes to poly(A) binding and HuR oligomerization. The hinge domain mediates nuclear–cytoplasmic shuttling. Domain boundaries and unstructured linker regions are indicated, RNP sites are marked in pink, phosphorylation sites and kinases in black, and other post-translational modifications in blue.

Human antigen R (HuR), also known as embryonic lethal abnormal vision-like protein 1 (ELAVL1), is a key post-transcriptional regulator that stabilizes mRNAs and modulates multiple post-transcriptional processes, enabling cells and tissues to dynamically respond to internal and external stimuli (13). Encoded by the ELAVL1 gene, HuR consists of three RNA recognition motifs (RRM1, RRM2, and RRM3), which are structurally almost identical despite considerable sequence divergence (**Figure 1**). RRM1 and RRM2 cooperate to bind AU-rich elements (AREs) located within the 3’ untranslated regions (3’ UTRs) of target mRNAs, thereby influencing mRNA stability and translational efficiency (14). RRM3 contributes to RNA-binding affinity and mediates protein–protein interactions. The hinge region (HNS) between RRM2 and RRM3 contains a nucleocytoplasmic shuttling sequence, which allows HuR to translocate between the nucleus and cytoplasm, thereby regulating mRNA stability and translation (15). Structural and computational assays using phosphorylated and phosphomimetic HuR proteins demonstrate that essential residues required for maintaining nuclear HuR are located at the N-terminal region of the HNS sequence, including R205, R206, and F207, as well as possibly H212, H213, R217, and R219 (15, 16).

It is a widely expressed RBP that exhibits multifaceted functions in cancer initiation and progression, primarily acting in an oncogenic capacity, while also displaying tumor-suppressive roles through the regulation of target genes (17, 18). Nuclear-cytoplasmic shuttling of HuR modifies its subcellular localization and regulates its functions in CRC. Studies have shown that expression levels of HuR significantly increased in the cytoplasm of CRC cells (19–21), and are closely associated with tumor invasion, metastasis, and poor prognosis in CRC patients (22). Meanwhile, the HuR protein can compete with non-coding RNAs (ncRNAs) or other RBPs for binding to the 3’UTR, thereby competitively or cooperatively regulating the progression of CRC. Despite these insights, the precise mechanisms by which HuR regulates the post-transcriptional process in CRC remain unclear. In this review, we first provide a comprehensive overview of the complex and multi-faceted mechanisms through which HuR regulates mRNA expression, controls the cell cycle, and modifies the tumor microenvironment in CRC. We also discussed the challenges in developing advanced HuR-targeted therapeutic strategies, offering valuable guidance for the development of therapies aimed at inhibiting CRC progression and metastasis.

## 2 HuR undergoes post-translational modifications

HuR undergoes various post-translational modifications (PTMs) that regulate its stability, localization, and interaction with target mRNAs, thereby influencing cellular processes such as cancer initiation and progression (23). HuR undergoes various PTMs, including phosphorylation, ubiquitination, methylation, and so on. These modifications influence HuR’s interactions with different proteins in complex protein networks, determining its subcellular localization and function.

### 2.1 HuR dynamically localizes by nucleo-cytoplasmic shuttling

HuR is primarily localized in the nucleus under normal conditions. Its export to the cytoplasm is considered a prerequisite for protecting homologous target mRNAs from rapid degradation. The stimulus-dependent translocation between the nucleus and cytoplasm, known as “HuR shuttling” is regarded as the initial and critical step in HuR-mediated mRNA stabilization in CRC. This process may also be closely linked to the general mRNA export pathway in higher eukaryotes (24).

Pathologically, increased cytoplasmic HuR abundance is a hallmark of various cancer types, making cytoplasmic HuR levels a potential prognostic indicator for poor survival outcomes in certain cancer patients. Similarly, in CRC development, HuR exerts significant regulatory effects (23). Studies have shown that in 76% of colorectal adenomas and 94% of CRC cases, HuR undergoes cytoplasmic translocation, which correlates with increased invasiveness, metastasis, and poor prognosis (25). Under physiological conditions, HuR is mainly located in the nucleus (26). However, upon exposure to various stressors, such as hypoxia, inflammation, radiation, or other stimuli, HuR binds to AU-rich sequences in the 3’ UTR of mRNA and stabilizes these target mRNAs (27–29). This interaction facilitates the mRNA’s export to the cytoplasm, where HuR protects it from exonuclease degradation and enhances translation. Afterward, HuR rapidly returns to the nucleus. The dynamic shuttling between the nucleus and cytoplasm is a key mechanism through which HuR executes its mRNA stabilization and translation functions (4). This phenomenon has drawn significant attention, highlighting HuR as a potential therapeutic target.

### 2.2 PTMs of HuR modulate its RNA-binding affinity and subcellular localization

HuR undergoes various PTMs, with phosphorylation being the most common. Modifications near RRMs typically affect its RNA-binding affinity, while those in the hinge region regulate subcellular localization. HuR’s phosphorylation is mediated by checkpoint kinase 2 (Chk2) and p38 mitogen-activated protein kinase (p38 MAPK), particularly at Ser88 and Thr118 (30), which are critical for the regulation of HuR’s mRNA splicing (28, 31). Similarly, phosphorylation at Ser318 by protein kinase Cδ (PKCδ) significantly enhances HuR stability, driving overexpression in CRC cells (32, 33). Consistent with this, Studies on HuR mutants show that phosphorylation-deficient mutants (e.g., S88A, S100A, T118A) hinder HuR’s interaction with TRA2β4 mRNA and its related functions, while phosphorylation-mimicking mutants (e.g., S88D, S100D, T118D) restore this interaction, underscoring the crucial role of phosphorylation in HuR function (34).

Ubiquitination is a PTM that marks proteins for degradation by the proteasome. HuR can be ubiquitinated, particularly under conditions where its activity needs to be tightly controlled, with β-TrCP serving as the E3 ligase responsible for HuR’s ubiquitination and degradation (35). In this context, lncRNA OCC-1 enhances the binding of β-TrCP to HuR, facilitating its degradation and suppressing HuR-driven oncogenesis in CRC (36). Under heat shock conditions, HuR undergoes proteasome-mediated degradation at lysine 182 (37). On another note, Co-activator-associated arginine methyltransferase 1 (CARM1/PRMT4) methylates arginine residue R217 in macrophages, human cervical cancer cells, and human embryonic stem cells, enhancing HuR’s stabilization of target gene mRNAs (38). Methylation of HuR in CRC, though less studied, has been shown to affect its ability to bind to target RNAs and may influence its role in regulating gene expression. This modification can play a role in cellular processes like stress response or cell cycle progression (39). E3 ubiquitin ligases play an important role in the biological functions of gastrointestinal tumors by participating in NEDDylation modification. Under the mediation of NEDD8 E3 ligase Mdm2, NEDDylation of HuR at K283, K313 and K326 promotes the stability of HuR protein and stabilizes its localization in the nucleus, which promotes the proliferation of colon cancer cells (40). During CoCl2-induced hypoxic stress, Caspases 3 and 7 cleave HuR at D226 in the cytoplasm, generating two HuR cleavage products (CPs). Among them, HuR-CP1 interacts with transportin 2 (Trn2) and is transported back to the nucleus (41, 42), which allow HuR to function as a key regulator of gene expression by controlling the stability, translation, and localization of target mRNAs.

## 3 HuR regulates the fate of mRNAs in CRC

HuR enhances the expression of genes involved in key processes like cell survival, proliferation, and metastasis by stabilizing their mRNAs (43). HuR also influences gene expression by regulating pre-mRNA alternative splicing, thereby promoting proliferation and invasiveness in CRC. Meanwhile, these regulatory activities of HuR are modulated by interactions with ncRNAs and RBPs, forming a complex gene regulatory network. Below are the key way in which HuR, through coordination with ncRNAs and Ribonucleoproteins(RNPs), promotes tumor progression through mRNA stabilization and pre-mRNA alternative splicing.

### 3.1 HuR stabilizes oncogenic mRNAs

By binding to mRNAs of pro-survival genes such as SIRT1, HuR prevents their RNase-mediated degradation (e.g., by CNOT7), thereby increasing mRNA stability and promoting the expression of these critical survival factors. In CRC, HuR stabilizes mRNAs such as MMP-9 (44), c-MYC (45), and BCL-2 (46), which play essential roles in regulating CRC cell proliferation, survival, and metastasis. These HuR-regulated genes contribute to the tumorigenic behavior of CRC cells, influencing both tumor progression and resistance to chemotherapy.

As a key post-transcriptional regulator of vascular endothelial growth factor (VEGF), HuR binds to VEGF mRNA and extends its half-life from less than 1 hour by 2.5–8-fold, thereby significantly enhancing VEGF production. This mechanism, combined with the observed upregulation of HuR under hypoxic stress, supports the hypothesis that HuR acts as a critical upstream mediator of tumor angiogenesis (22). Cyclooxygenase-2 (COX-2) expression is fundamental among HuR-regulated oncogenic transcripts. Nuclear expression of HuR is present in 98% of CRC tissues; 53% of cases show additional cytoplasmic expression (47). The cytoplasmic localization of HuR is significantly correlated with COX-2 expression levels and advanced tumor stages and poor clinical prognosis, suggesting that HuR overexpression may promote the progression of colon cancer by stabilizing COX-2 mRNA. Cytoplasmic HuR directly binds to the AU-rich elements (AREs) in the 3’ UTR of COX-2 mRNA, shielding it from degradation mediated by microRNAs such as miR-16 (48). This interaction stabilizes COX-2 mRNA and promotes tumor angiogenesis and CRC progression by promoting VEGF production (49).

Nevertheless, some studies have found that HuR can also exhibit adverse regulatory effects, such as inhibiting growth and metastasis and regulating cell contact inhibition (48). It has also been found that the knockdown of HuR reduces COX-2 mRNA levels but does not significantly affect protein levels (50). Moreover, it has also been reported to stabilize p53 and p21 transcripts, suggesting that HuR may act as either an oncogene or a tumor suppressor (51). These divergent outcomes are largely determined by HuR’s subcellular localization, post-translational modifications, and interacting noncoding RNAs, which together shape its mRNA target repertoire. Overall, HuR functions as an oncogene in CRC, while its potential tumor-suppressive roles require further investigation to be confirmed.

### 3.2 HuR modulates Pre-mRNA alternative splicing

Since the discovery of pre-mRNA splicing by Chow et al. in 1977 (52), it has become clear that pre-mRNA maturation requires the removal of introns and joining exons, which is known as RNA splicing, includes constitutive and alternative splicing (AS) (49, 53). It affects approximately 95% of gene expression (54). As an RBP, HuR plays a significant role not only in the stability and translation of mature mRNA in CRC but also in influencing pre-mRNA processing, particularly alternative splicing, to enhance gene expression diversity (55).

In CRC cells, HuR binds to pre-mRNA, especially near intronic or exonic regions, and affects the alternative splicing process. For example, HuR binds to the exon 2a region of TRA2β gene pre-mRNA, regulating its alternative splicing and promoting the generation of TRA2β4 mRNA, which contains multiple premature termination codons. Excessive TRA2β4 expression inhibits gene expression, promoting cell proliferation and revealing HuR’s potential oncogenic function in CRC through AS regulation. The silencing of HuR or inhibition of the Chk2/p38 MAPK pathway, which phosphorylates HuR to enhance its mRNA-binding affinity and cytoplasmic translocation, effectively inhibits the production of TRA2β4 (56). Moreover, deleting the 39-nucleotide region near exon 2 of TRA2β further impedes this regulatory mechanism, confirming the critical role of HuR in AS regulation (34). Additionally, HuR influences CRC invasiveness by regulating novel tight junction protein 1 (ZO-1) AS patterns. Glioma suppressor candidate gene 1 (GLTSCR1) reduces ZO-1 transcription elongation, providing a time window for HuR to bind with specific sequences in ZO-1 intron 22 and spliceosome recognition sites in exon 23, thus promoting the inclusion of exon 23. The inclusion of exon 23 inhibits migration and invasion of CRC cells (57).

### 3.3 HuR, ncRNA, and other RBPs constitute a gene regulatory network

The expression levels of ncRNAs are closely associated with CRC progression and metastasis (58–60). Recent functional studies indicate that HuR and microRNAs (miRNAs) may share the same mRNA functional sites (20, 21, 61–63). At each stage of CRC, many miRNAs exhibit altered expression, interact with HuR, and participate in regulating CRC cancer markers (64–67). Fengxing Huang et al. discovered that HuR promotes abnormal lipid accumulation and tumor growth in CRC cells by stabilizing VDR mRNA through direct binding and counteracting the inhibitory effect of miR-124-3p, thereby regulating triglyceride and cholesterol metabolic homeostasis (20). From the tumor microenvironment perspective, Antonio Biondi et al. demonstrated that HuR promotes CRC cell proliferation by stabilizing HOXC6 mRNA and enhancing its transcriptional activity, while regulating the molecular network of miR-34b-5p/SNHG3 mediated by CAFs-derived extracellular vesicles (68).We summarize the current research on the mechanisms by which ncRNAs interact with HuR to regulate tumor-related cellular processes in CRC (**Table 1**) and will subsequently elaborate on the therapeutic applications of miRNA- and lncRNA-mediated HuR targeting.

**Table 1:** ncRNAs involved in HuR-mediated post-transcriptional regulation in CRC.

Type	ncRNAs	Level	Mechanisms	Reference
circRNA	circAGO2	up	miR-224-5p and miR-143-3p, located near the ARE of oncogenes, regulate proto-oncogenes such as HNF4, NOTCH4, and SLC2A4. Along with miR-1-3p, they increase the expression of RBBP4, promoting the proliferation and invasion of CRC cells.	(69–71)
circRNA	circPPFIA1, miR-155-5p	down	circPPFIA1-L and circPPFIA1-s function as sponges for miR-155-5p, influencing tumor suppressor genes and oncogenes.	(21)
circRNA	circ0104103, miR-373-5p	down	Circ0104103 functions as a competitive endogenous RNA (ceRNA) for miR-373-5p, reducing HuR expression and inhibiting CRC progression.	(65)
circRNA	circNOLC1, miR-212-5p	up	CircNOLC1 sponges miR-212-5p, promoting HuR-mediated upregulation of c-Met, and regulates the reprogramming of the oxidative pentose phosphate pathway to facilitate hepatic metastasis of CRC.	(72)
miRNA	miR-519	down	Downregulating HuR by targeting its coding region exerts tumor-suppressive effects.	(66, 73–75)
miRNA	miR-16	down	HuR reduces miR-16 levels; in cancer cells, miR-16 expression diminishes COX-2 expression and prostaglandin synthesis.	(5, 48, 76)
miRNA	miR-324-5p	down	Targeting HuR limits cellular proliferation and migration.	(77, 78)
miRNA	miR-22	down	By targeting the 3’-UTR of HuR and directly binding to it, HuR expression is inhibited, suppressing CRC cell growth.	(64, 67)
miRNA	miR-31	down	Downregulates cyclins and VEGF, inhibiting proliferation and angiogenesis in CRC.	(79, 80)
miRNA	miR-122	up	Accelerates extracellular vesicle (EV)-mediated export of miR-122 and enhances stress responses, which are associated with the poor prognostic subtype of metastatic CRC.	(62, 81, 82)
miRNA	miR-548c	down	Inhibits HuR-mediated mRNA stability, suppressing CRC progression.	(83)
miRNA	miR-494	up	By downregulating HuR and targeting APC, it induces Wnt/β-catenin signaling, thereby promoting CRC cell growth.	(84–86)
miRNA	miR-34	down	The miR-34 family is an important tumor-suppressor miRNA due to its synergistic effect with the tumor suppressor gene TP53.	(87)
miRNA	miR-96	up	Binds to HuR, stabilizing HuR.	(88, 89)
miRNA	miR-194	up	miR-194 antagonistically regulates nucleolin expression with HuR, promoting cell migration and invasion in CRC cells.	(90, 91)
miRNA	miR-34b-5p	up	miR-34b-5p targets HuR; it competes with HuR for binding to OIP5-AS1, thereby inhibiting OIP5-AS1, the PI3K/Akt pathway, and CRC progression.	(87, 92)
lncRNA	OCC-1	down	Enhances the binding of HuR to β-TrCP1, leading to HuR ubiquitination and degradation, thus inhibiting cell growth.	(36)
lncRNA	GMDS-AS1	up	Binds to HuR, preventing its ubiquitination, stabilizing STAT3 mRNA, activating the STAT3/Wnt pathway, and promoting CRC development.	(93)
lncRNA	SPRY4-IT1	up	Enhances the interaction between HuR and tight junction protein mRNAs, promoting the expression of claudin-1, claudin-3, occludin, and JAM-1.	(94)
lncRNA	OIP5-AS1, miR-34b-5p	up	Binds to HuR, competes with miR-34b-5p, stabilizes HuR, and supports CRC progression.	(92)
lncRNA	TNFRSF10A-AS1, miR-3121-3p	up	Acts as a sponge for miR-3121-3p, leading to HuR upregulation and promoting CRC progression.	(95)

miRNA/miR, microRNA; circRNA, circular RNA; lncRNA, long non-coding RNA; CRC, colorectal cancer; HuR, Human Antigen R; ARE, AU-rich element; RBBP4, Retinoblastoma Binding Protein 4; ceRNA, competing endogenous RNA; EV, extracellular vesicle; VEGF, vascular endothelial growth factor; COX-2, cyclooxygenase-2; STAT3, signal transducer and activator of transcription 3; APC, adenomatous polyposis coli; β-TrCP1, β-Transducin Repeat Containing E3 Ubiquitin Protein Ligase 1; JAM-1, junctional adhesion molecule 1; PI3K/Akt, phosphatidylinositol 3-kinase/protein kinase B; TP53, tumor protein p53; UTR, untranslated region; c-Met, cellular-mesenchymal epithelial transition factor; PPP, pentose phosphate pathway; GMDS-AS1, GMDS antisense RNA 1; SPRY4-IT1, SPRY4 intronic transcript 1; OIP5-AS1, OIP5 antisense RNA 1; TNFRSF10A-AS1, TNFRSF10A antisense RNA 1; OCC-1, overexpressed in colon carcinoma 1.

In regulating mRNA stability, HuR functions not only in coordination with ncRNAs but also interacts with other RBPs to form a complex regulatory network (96, 97). HuR competitively binds to different RBPs at different nonoverlapping or common sites and antagonistically regulates mRNA stability and translation. For example, HuR and AUF1 competitively bind p21 and Cyclin D1 mRNAs, which HuR stabilizes, whereas AUF1 promotes mRNA degradation, inhibits mRNA expression, and hinders cell cycle progression (98, 99). Similarly, HuR competes with CUGBP1 for binding occludin and E-cadherin mRNAs. Antagonistic to HuR, CUGBP1 promotes mRNA degradation and inhibits cell proliferation and adhesion (100). HuR also antagonizes RBPs such as TIAR and TTP and affects the expression of pro-proliferative and anti-apoptotic genes such as JunD (101). On the other hand, in terms of co-regulation, a few RBPs cooperate with HuR to improve the stability and translation efficiency of the target mRNA. For example, hematopoietic zinc finger protein (Hzf) cooperates with HuR to regulate p53 expression (51). Moreover, recent studies have shown that certain RBPs and miRNAs can co-target the same mRNA to regulate its expression in a competitive or synergistic manner (102–104). This multi-layered regulatory mechanism further complicates the fine-tuning of gene expression, making HuR-targeted regulatory effects even more challenging.

**Figure 2:**
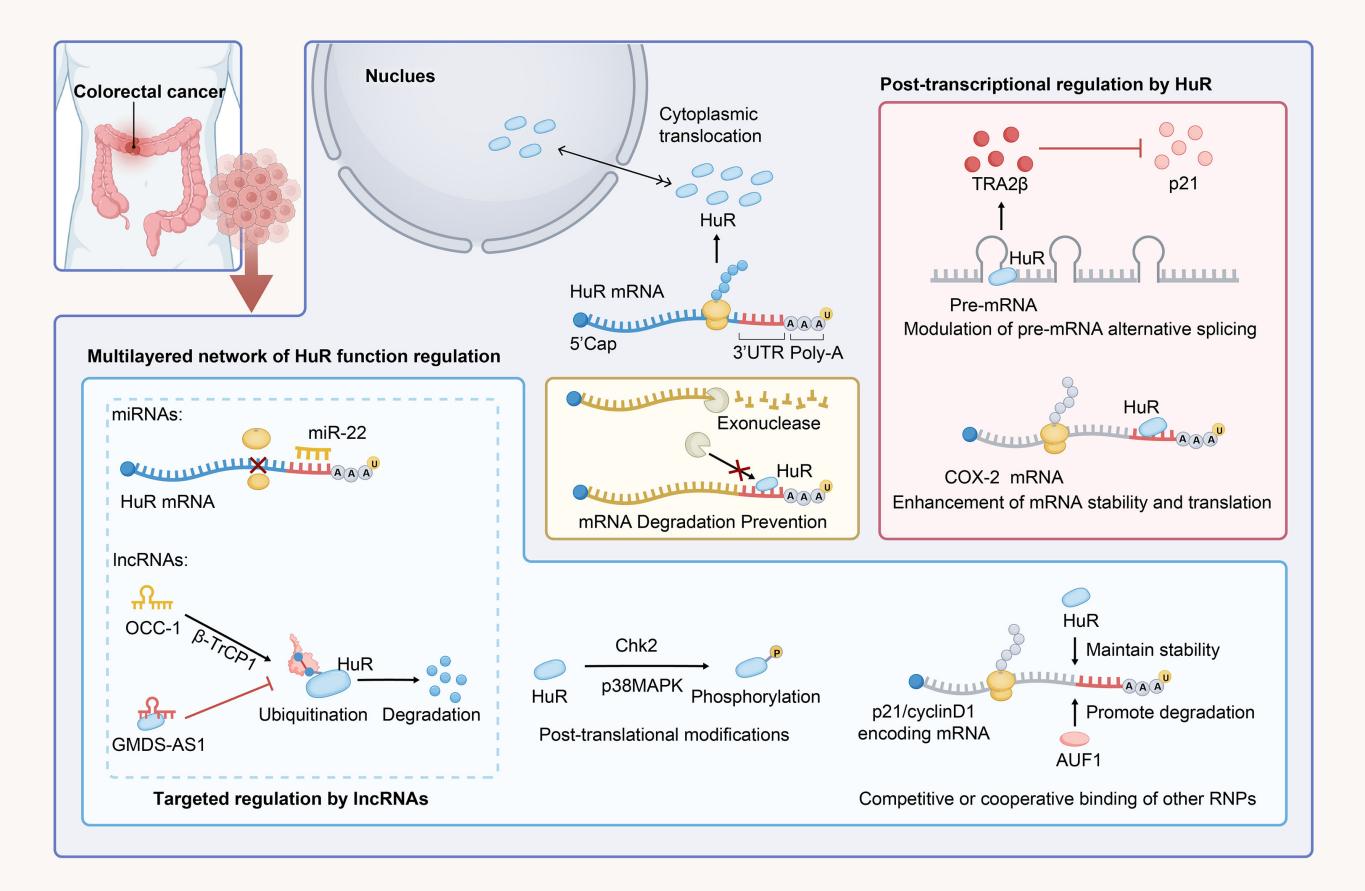
HuR-mediated regulation of CRC through mRNA stabilization and translational modulation. In normal cells, HuR is predominantly localized in the nucleus; however, under stimuli such as CRC, it dynamically translocates to the cytoplasm. HuR binds to AU-rich sequences in the 3’ UTR of mRNAs and stabilizes them, preventing exonuclease-mediated degradation of target mRNAs. It also regulates gene expression by modulating alternative splicing of pre-mRNA. The stabilization and regulatory effects of HuR on mRNA are influenced by various factors, including phosphorylation, while ncRNAs and other RBPs can compete with HuR, either inhibiting or synergizing with its regulatory functions.

HuR promotes CRC progression by mediating post-transcriptional gene expression through nucleocytoplasmic shuttling, cytoplasmic mRNA stabilization, splicing regulation, and phosphorylation-dependent interactions with ncRNAs and RBPs, as summarized in **Figure 2**. Nevertheless, significant gaps remain in understanding the molecular determinants of HuR-mediated oncogenic mRNA recognition, particularly how cellular stressors such as hypoxia, inflammation, and metabolic stress induce HuR translocation from the nucleus to the cytoplasm. From a therapeutic perspective, whether pharmacological disruption of HuR-RNA interactions induces compensatory activation of other RBPs, such as AUF1, is a crucial consideration for precision-targeting strategies. In the future, further investigation is needed to explore the role of HuR in stabilizing mRNA in CRC.

## 4 HuR influences several cancer traits of CRC

HuR initiates an extensive cell survival program by increasing mRNAs’ stability, encoding key cell cycle regulators and anti-apoptotic factors (105). It drives cell cycle progression, promotes anti-apoptotic proteins’ expression, and inhibits pro-apoptotic proteins’ expression. *In vitro* studies have demonstrated that HuR regulates gene expression in essential cellular processes, associated with involvement in the cell cycle, cell death, proliferation, and differentiation.

### 4.1 HuR modulates cell proliferation and survival

HuR plays a vital role in cell cycle regulation, primarily by stabilizing mRNAs of cell cycle-related genes and promoting their translation, thereby driving the cell cycle and accelerating CRC cell proliferation. Reducing HuR levels (such as in RKO cells expressing HuR antisense RNA) significantly inhibits cell growth (106, 107). HuR’s subcellular localization is closely related to its function, and cytoplasmic levels peak during the S-phase and G2/M phase of the cell cycle. Under stress conditions, the increased cytoplasmic HuR stabilizes the mRNAs of Cyclin A and Cyclin B1 (106), promoting the expression of pro-oncogenic genes such as c-Myc (105).

High expression of c-Myc in CRC cells contributes to the maintenance of cell proliferation. HuR, as a critical pro-proliferative factor, promotes the proliferation of CRC cells by enhancing the stability of c-Myc mRNA and promoting its high expression (45). By prolonging the half-life of c-Myc mRNA, HuR delays its degradation, enhances its translation expression, and promotes the proliferation of CRC cells (108, 109). Furthermore, HuR can bind to and stabilize multiple cyclin mRNAs, such as Cyclin A2, Cyclin B1 (106, 110), Cyclin D1 (111), and Cyclin E1 (76), which promote the transition from G1 to S phase and accelerate cell cycle progression in CRC cells (106). HuR also binds to the 3’ UTR of CDC6 mRNA, prolonging its half-life and increasing the expression of CDC6 protein as an essential regulator of DNA replication (112). CDC6 promotes cell cycle progression by activating the CDK2-Cyclin A/E complex that binds to p21 or p27 (46). HuR also inhibits the expression of p21, deregulates the negative regulation of CDKs (such as CDK2) and Cyclin A, and promotes the transition of cells from the G1 phase to the S phase (113). By promoting c-Myc expression, HuR indirectly downregulates p21, further enhancing CDK2 and Cyclin A activity, thereby promoting cell cycle progression (114). Through these mechanisms, HuR orchestrates the upregulation of cyclins and the suppression of p21 (115), collectively driving CRC cell proliferation and tumor development.

Experimental studies have shown that HuR plays a key role in tumor cell survival. In conditional HuR knockout mice (HurIKO), tumor burden is significantly reduced, with intestinal tumor numbers decreasing by approximately 60% and total tumor area decreasing by about 70% in CRC, alongside a notable reduction in tumor volume (116). These changes are directly related to increased apoptosis and decreased proliferation (107). In HuR-deficient tumor tissues, apoptosis increased fivefold, while cell proliferation significantly decreased, indicating that HuR deficiency activates apoptotic pathways and reduces tumor burden (117). HuR regulates the expression of anti-apoptotic genes, enhancing the survival of CRC cells. It prolongs the half-life of anti-apoptotic factors like Bcl-2 and Cyclin D1 (111), maintaining their stable protein expression and preventing cells from entering apoptotic pathways. Furthermore, HuR also regulates the expression of CDC6, inhibiting the activation of Apaf-1 and preventing the formation of apoptotic bodies (112), further enhancing cell survival. For example, in human colorectal adenocarcinoma SW480 cells, inhibition of HuR and C2ORF68 results in increased expression of the pro-apoptotic factor Bax and decreased expression of anti-apoptotic factors Bcl-2, c-Myc, Cyclin D, and Cyclin A, promoting cell apoptosis. This indicates that HuR, in coordination with C2ORF68, regulates the expression of apoptosis-related genes, directly affecting CRC cell apoptosis and survival (118).

Moreover, HuR helps CRC cells survive under stress conditions by binding to and stabilizing mRNAs related to cell survival, such as Bcl-2 (119) and XIAP (120), to prevent apoptosis. In HuR knockout mice, the mRNA expression of anti-apoptotic factors Sirt1 and VEGF is significantly downregulated (121). In contrast, the mRNA expression of pro-apoptotic factors Tp53, Caspase-9, and Fas is significantly upregulated, activating the p53-dependent intrinsic apoptotic pathway (116). This is considered the main reason for the reduced tumor burden caused by HuR deficiency (107). Survivin is another key anti-apoptotic factor; binding of HuR to its mRNA increases its stability and expression level, inhibiting cell apoptosis (122). Particularly in p53-deficient cells, HuR significantly enhances tumor cell survival and drug resistance by regulating Survivin. It should be noted that under prolonged or severe stress, HuR may instead exert a pro-apoptotic effect (123).

### 4.2 HuR affects cancer cell metabolism

Recent studies have revealed that HuR plays a key role in lipid metabolism and tumor development in CRC. Studies have found that HuR promotes the expression of the vitamin D receptor (VDR) by increasing triglyceride (TG) and total cholesterol (TC) levels in CRC cells, thereby maintaining lipid homeostasis. Experimental data show that HuR overexpression directly binds to the coding sequence (CDS) and 3’UTR of VDR, thereby indirectly increasing VDR levels by inhibiting miR-124-3p. Further evidence from xenograft models confirms that targeting HuR can suppress VDR expression, reduce TG and TC production, and thus slow CRC growth. These results suggest that the HuR/miR-124-3p/VDR axis may regulate metabolism by modulating CRC lipid homeostasis (20).

**Figure 3:**
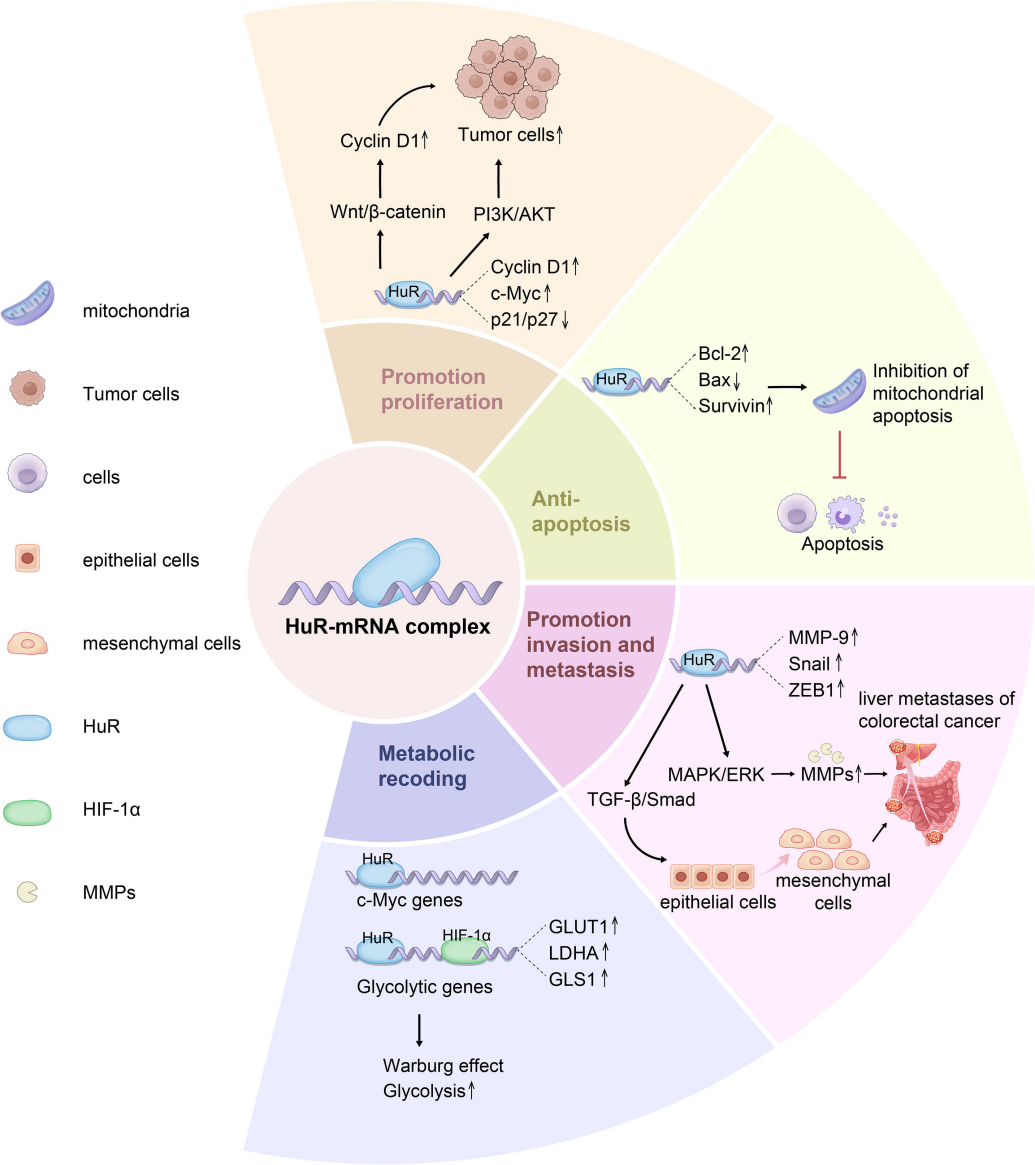
HuR promotes tumor cell phenotype including cell proliferation, survival and metabolism of CRC. HuR is a key RNA-binding protein that regulates the post-transcriptional expression of numerous genes involved in cell proliferation, survival, and metabolism. HuR enhances the stability of Cyclin D1, c-Myc, and VEGF mRNAs, promoting cell proliferation and accelerating cell cycle progression via the Wnt/β-catenin pathway. Additionally, HuR suppresses the translation of apoptosis-related genes Bcl-2, Bax, and Survivin, thereby enhancing tumor cell survival and inhibiting mitochondrial pathway-mediated Caspase-9/3 activation. Furthermore, HuR facilitates the nuclear export of MMP-9, Snail, and ZEB1 mRNAs, upregulating EMT-related transcription factors, downregulating E-cadherin, and upregulating N-cadherin, ultimately promoting tumor invasion and metastasis. In metabolic regulation, HuR stabilizes the mRNAs of key glycolytic enzymes GLUT1, LDHA, and PKM2, enhancing glycolysis and reinforcing tumor metabolic adaptation via the Warburg effect.

HuR enhances cell cycle progression, supports tumor survival under stress, and maintains lipid homeostasis, sustaining CRC growth and enabling tumor adaptation to changing conditions (**Figure 3**). Although it is known that HuR influences tumor metabolism by regulating the mRNA stability of metabolism-related genes, the specific mechanisms are not yet clear. For example, does HuR participate in CRC metabolic reprogramming through lactylation modification, affecting the metabolic end product lactate that is closely related to glycolysis? Additionally, does HuR regulate CRC cell proliferation and apoptosis through pathways such as ferroptosis and necroptosis? These mechanisms still need further exploration. Furthermore, whether HuR’s effects on growth, proliferation, and apoptosis differ at various stages of CRC progression (from early to late stages) remains to be determined, which will be crucial for developing targeted therapeutic strategies.

## 5 HuR orchestrates the tumor microenvironment in CRC

In the process of CRC occurrence and development, in addition to altered stability and expression of mRNAs in tumor cells, changes in the diverse relationships between different types of cells in the TME are essential (12). The effect of HuR on the genetic variation of CRC directly regulates CRC cell behavior and influences the reorganization and functional changes of molecular and intercellular interactions (124). Our previous review summarized these data and showed that RBPs play a critical role in TME formation by inducing inflammation (125, 126), immunity (127), ECM remodeling (128, 129), and vasculature during CRC progression (12, 130). Accumulating evidence suggests that components of the TME, such as immune cells, cytokines, inflammatory factors, extracellular matrix (ECM), and vasculature, can promote CRC development by inducing immunosuppression and altering the TME (131). The perspective of cancer research has shifted from focusing solely on cancer cells to emphasizing the importance of the TME. Based on the tumor-promoting role of HuR in the TME, various RBP-TME CRC therapeutic compounds have been developed, including small molecule inhibitors (such as antisense oligonucleotides (ASOs)), gene manipulation, targeted delivery of Small interfering RNA (siRNA), agonists, and tumor vaccines (12, 21), providing a cutting-edge perspective for the clinical treatment of CRC.

### 5.1 HuR influences inflammatory signaling in TME

In the inflammatory microenvironment of CRC, HuR, as a key RBP, is deeply involved in the regulation of inflammatory responses and significantly affects CRC development. HuR can stabilize the mRNAs of pro-inflammatory cytokines, increase their stability and translation efficiency, aggravate the inflammatory microenvironment, and promote CRC progression. The long-term inflammatory state also alters the TME and promotes CRC development. Yiakouvaki et al. demonstrated that myeloid cell-specific HuR deficiency in mice enhances inflammation, with increased production of pro-inflammatory cytokines by myeloid-derived cells, particularly macrophages. Conversely, overexpression of HuR in bone marrow-derived myeloid cells has been shown to attenuate colitis by restraining excessive cytokine production, thereby suppressing colitis-associated CRC (132). In contrast, bone marrow overexpression of HuR correspondingly suppresses colitis and CRC. HuR in CRC cells stabilizes ARE-containing RNAs and/or promotes their translation, enhancing the production and release of oncogenic factors such as COX-2 (133), tumor necrosis factor-alpha (TNF-α) (134), interleukin-8 (IL-8) (135, 136) and interleukin-6 (IL-6) (137), enhancing their roles in the inflammatory microenvironment, exacerbating the inflammatory response, and promoting CRC cells proliferation and survival (36). Furthermore, HuR modulates the expression of critical inflammatory mediators such as CCL2 and CCR2, forming a HuR/CCL2/CCR2 axis that governs macrophage migration, infiltration, and retention at sites of inflammation (132, 138). Under chronic inflammatory conditions, macrophages release cytotoxic molecules that induce DNA damage in epithelial cells, drive the formation of precancerous lesions, and ultimately promote the development of CRC (139).

Although HuR is generally regarded as a stabilizer of pro-inflammatory mRNAs in CRC progression, studies have shown that under certain conditions, HuR can also play a negative regulatory role by inhibiting the translation of specific inflammatory mRNAs through mechanisms such as transcript sequestration or recruitment of repressive complexes. For example, overexpression of HuR in mouse macrophages blocks the translation of specific inflammatory mRNAs, demonstrating its potential to inhibit pathological inflammation. This dual regulatory function makes HuR a complex and potential therapeutic target in CRC (132). How can the dual pro-inflammatory and anti-inflammatory roles of HuR in the tumor microenvironment be explained? Does this bidirectional regulation depend on intracellular signaling states or interactions with other factors? Beyond the known regulatory mechanisms, could there be additional yet unidentified pathways or interacting proteins through which HuR modulates inflammation evasion in the tumor microenvironment? It remains unclear and requires further studies to be confirmed. Current studies have focused on inhibiting the RNA-binding activity of HuR to reduce its pro-inflammatory effects in CRC (15). However, given the diversity of HuR functions in different pathological settings, future research should aim to develop drugs that can precisely regulate HuR function to both block its pro-cancer effects and maintain or enhance its anti-inflammatory potential in CRC. This highlights the challenge of targeting HuR to achieve specific therapeutic effects.

### 5.2 HuR induces an immune-suppressive TME in CRC

HuR plays a crucial role in inducing and maintaining an immune-suppressive microenvironment in cancer. It achieves this by interacting with a series of immune cells such as MDSCs, Tregs, and macrophages. HuR enhances the infiltration of CD4^+^ T cells, including Th1 and cytotoxic effector subsets (140, 141), facilitates the reprogramming of functional pathways (139), and promotes the differentiation of Th17 cells (141). HuR also regulates the alternative splicing of genes involved in DNA deamination, thereby protecting germinal center (GC) B cells from DNA damage and apoptosis (142). Numerous studies have demonstrated that HuR exhibits distinct regulatory effects on macrophage polarization across multiple cancer types, such as glioblastoma (140), hepatocellular carcinoma (143), and lung cancer (144). However, its specific mechanisms in regulating immune cells within CRC remain largely unexplored.

CRC is typically characterized by dense infiltration of immune and inflammatory cells that produce cytokines (145). As the tumor progresses, tumor-associated macrophages (TAMs) respond to the hypoxic tumor microenvironment by secreting factors that promote immunomodulation, thereby facilitating tumor growth. TNF-α and IL-6 are key inflammatory cytokines involved in immune suppression and tumorigenesis in CRC, providing growth and expansion signals to tumor progenitor cells and enhancing CRC development. Studies have shown that HuR promotes chronic inflammation by stabilizing TNF-α and IL-6 mRNAs and enhancing their expression within the tumor microenvironment (TME) (132, 146), thereby suppressing anti-tumor immune responses. However, HuR plays a dual role in inflammation-driven immune responses in CRC. Some studies have reported that HuR overexpression can also inhibit the translation of selective inflammatory mRNAs, suggesting that HuR may act as a negative regulator of pathological inflammation (147), thereby mitigating intestinal inflammation and CRC progression (132, 146). For instance, Lang et al. found that IL-18 expression was predominantly downregulated by the HuR inhibitor MS-444, which decreased IL-18 mRNA and protein levels in LPS-stimulated macrophages in CRC. On one hand, IL-18 activates Th1 and Th17 responses to drive immune activation; on the other, it enhances intestinal barrier integrity and regeneration to protect against microbial invasion. Thus, downregulation of IL-18 may impair T cell responses to DSS-induced injury and compromise epithelial regeneration and proliferation. Consequently, HuR inhibition can reduce eosinophil recruitment into tumors and increase CRC tumor size and invasiveness, which is closely associated with poorer CRC prognosis (146).

HuR has also been implicated in modulating the expression of immune checkpoint molecules, especially Programmed Cell Death Ligand 1 (PD-L1) (148). This immune checkpoint molecule is critical for immune evasion by CRC cells. HuR may help stabilize the mRNA of PD-L1, increasing its expression on the surface of CRC cells, thus contributing to immune escape. The overexpression of PD-L1 directly inhibits CD8+ T cell activation and cytotoxic function while potentially increasing the immunosuppressive activity of Tregs, further reinforcing the immunosuppressive TME (137). At the post-transcriptional level, HuR upregulates the expression of immune evasion-related genes, enabling CRC cells to evade immune surveillance and facilitating tumor progression. CRISPR/Cas9-mediated knockout of HuR in MDA-MB-231 triple-negative breast cancer cells resulted in altered expression of mRNAs involved in immune evasion pathways, including autophagy, T-cell costimulation, TCR signaling, and TGF-β signaling (149). These findings highlight HuR’s critical role in integrating post-transcriptional regulatory mechanisms to shape the TME, drive immune evasion, and promote CRC progression, making it a promising therapeutic target.

### 5.3 HuR mediates extracellular matrix remodeling

The ECM is a heterogeneous and vital component of the TME, composed of water, signaling molecules, and enzymes. The ECM contains various cytokines that mediate different signaling pathways and affect normal or tumor cells’ division, proliferation, and death (150). In tumorigenesis, the ECM plays an important and complex role in the formation of the colorectal TME (131). Disrupted ECM plasticity causes hydrolysis of proteins and other components at the primary tumor site, promoting the dissociation of cancer cells from their initial location and migration to different parts of the body. These formed microenvironments are also called pre-metastatic niches (PMNs), where various cytokines such as VEGF and growth factor-beta (TGF-β) are recruited in the ECM (151).

Downregulation of E-cadherin is a hallmark of epithelial-mesenchymal transition (EMT) and is closely associated with tumor invasion and metastasis. HuR directly influences EMT by stabilizing E-cadherin mRNA and may indirectly regulate N-cadherin expression (152). Like E-cadherin, Matrix metalloproteinases (MMPs) are also characteristic ECM components of cancer and are considered potential diagnostic and prognostic markers of CRC. They promote tumor cell invasion and metastasis by degrading the ECM and altering its stiffness and mechanical properties (153). MMP-2 and MMP-9, as the major MMPs, degrade cell adhesion molecules (CAMs) in the ECM, such as integrins and fibronectin (FN1), and regulate cell adhesion and signaling with the ECM, which promotes CRC cell invasion and metastasis (44, 154). In addition, urokinase-type plasminogen activator (uPA), which is derived from the serine protease system, and its receptor uPAR, are involved in extracellular matrix degradation and the regulation of CRC cell migration, especially in advanced colon tumors. uPA and uPAR expression is up-regulated in advanced colon tumors, which affects tumor prognosis. uPA and uPAR are post-transcriptionally up-regulated by HuR in CRC cells via ARE-dependent mRNA stabilization, which promotes CRC cell uPA/uPAR pathway activity, ECM degradation, and migration (155).

### 5.4 HuR participates in EV packaging and secretion

Extracellular vesicles (EVs) include exosomes, microvesicles, and apoptotic bodies. They carry biomolecules such as DNA and RNA and play crucial roles in tumorigenesis, therapeutic response, and immune regulation (156). In CRC, EVs are essential mediators of signal transduction in the TME, influencing the function of surrounding cells through the RNA molecules they transport. EVs induce reprogramming of the TME around CRC cells, conferring immune escape ability to cancer cells and promoting tumor progression and metastasis (157). CRC cells are usually in a hypoxic and acidic microenvironment due to high oxygen consumption and accumulation of metabolic wastes. This stressful microenvironment enables cancer cells to evade immune surveillance and resist chemotherapy-induced cytotoxicity (158). Under these conditions, CRC cells accelerate the release of EVs. Cancer-associated fibroblasts (CAFs) promote CRC progression and chemoresistance via extracellular vesicle (EV)-mediated transfer of lncRNAs, but by distinct mechanisms. CAF-derived EVs deliver SNHG3 into CRC cells, where SNHG3 sponges miR-34b-5p to upregulate HuR and thereby enhance HuR’s association with HOXC6, increasing HOXC6 expression and driving tumor cell proliferation (159). Separately, CAF-derived exosomal CCAL interacts with HuR to augment HuR activity and cytoplasmic accumulation, leading to stabilization of β-catenin mRNA, activation of Wnt/β-catenin signaling, suppression of apoptosis, and promotion of oxaliplatin resistance (159). These findings highlight CAF-derived non-coding RNAs as potential therapeutic targets for overcoming chemotherapy resistance in CRC (68, 160).

As an RBP, HuR influences mRNA stability and translation, is involved in RNA molecule packaging and secretion in EVs, and regulates EV function. Interfering with HuR-associated EVs or their molecular targets, by disrupting EV secretion and uptake or blocking their interactions, may help overcome drug resistance, presenting a potential strategy for CRC therapy (158). Furthermore, studies have shown that HuR is overexpressed in CRC cells and is delivered to distant lung bronchial epithelial cells via exosomes (161). Upon uptake by recipient cells, HuR stabilizes specific mRNAs (such as c-Myc mRNA), enhancing their expression and activating cell proliferation-related signaling pathways. This supports tumor spread and promotes cell proliferation, migration, and invasion (162). To meet the nutritional demands of rapid growth, CRC cells promote angiogenesis, potentially utilizing EVs to facilitate this process (159).

### 5.5 HuR stimulates tumor angiogenesis and lymphangiogenesis in CRC

Tumor angiogenesis is a key characteristic of TME. In CRC, the endocrine function of endothelial cells (ECs) can activate the Notch signaling pathway, enhance cancer stem cell phenotypes, and further promote tumor progression (163, 164). Multiple pro-angiogenic cytokines, including VEGF, granulocyte-macrophage colony-stimulating factor (GM-CSF), tumor necrosis TNF-α, transforming TGF-β, chemokine CXCL1, IL-6, and IL-8, are involved in neovascularization in CRC (165). The mRNAs of these cytokines are typically unstable. HuR upregulates their expression by binding to AREs in the 3’UTRs of their mRNAs, enhancing mRNA stability and prolonging their half-lives. This post-transcriptional regulation plays a vital role in tumor angiogenesis (166). As an RNA-stabilizing factor, HuR overexpression increases angiogenesis-related proteins such as VEGF and hypoxia-inducible factor-1 (HIF-1), promoting local angiogenesis and the development of CRC (100).

HuR overexpression in CRC not only mediates angiogenesis but is also closely associated with lymphangiogenesis in the mesenchyme of tumor tissues. These tumor-associated lymphatic vessels may serve as conduits for tumor dissemination through the lymphatic system and are critical for tumor growth and aggressiveness (100, 167). HuR is involved in tumor-associated lymphangiogenesis through post-transcriptional mechanisms, potentially mediating lymphangiogenesis in the TME by influencing the expression of growth factor ligands and receptors, such as insulin-like growth factors 1 and 2 (IGF-1 and IGF-2) (168). This has important implications for understanding tumor progression and metastasis. Given the key role of HuR in tumor-associated angiogenesis and lymphangiogenesis, HuR may serve as an anti-angiogenic target and predictive marker in CRC therapy (131, 163). Further studies are needed to explore the specific mechanisms of HuR action in the TME to develop new therapeutic strategies.

**Figure 4:**
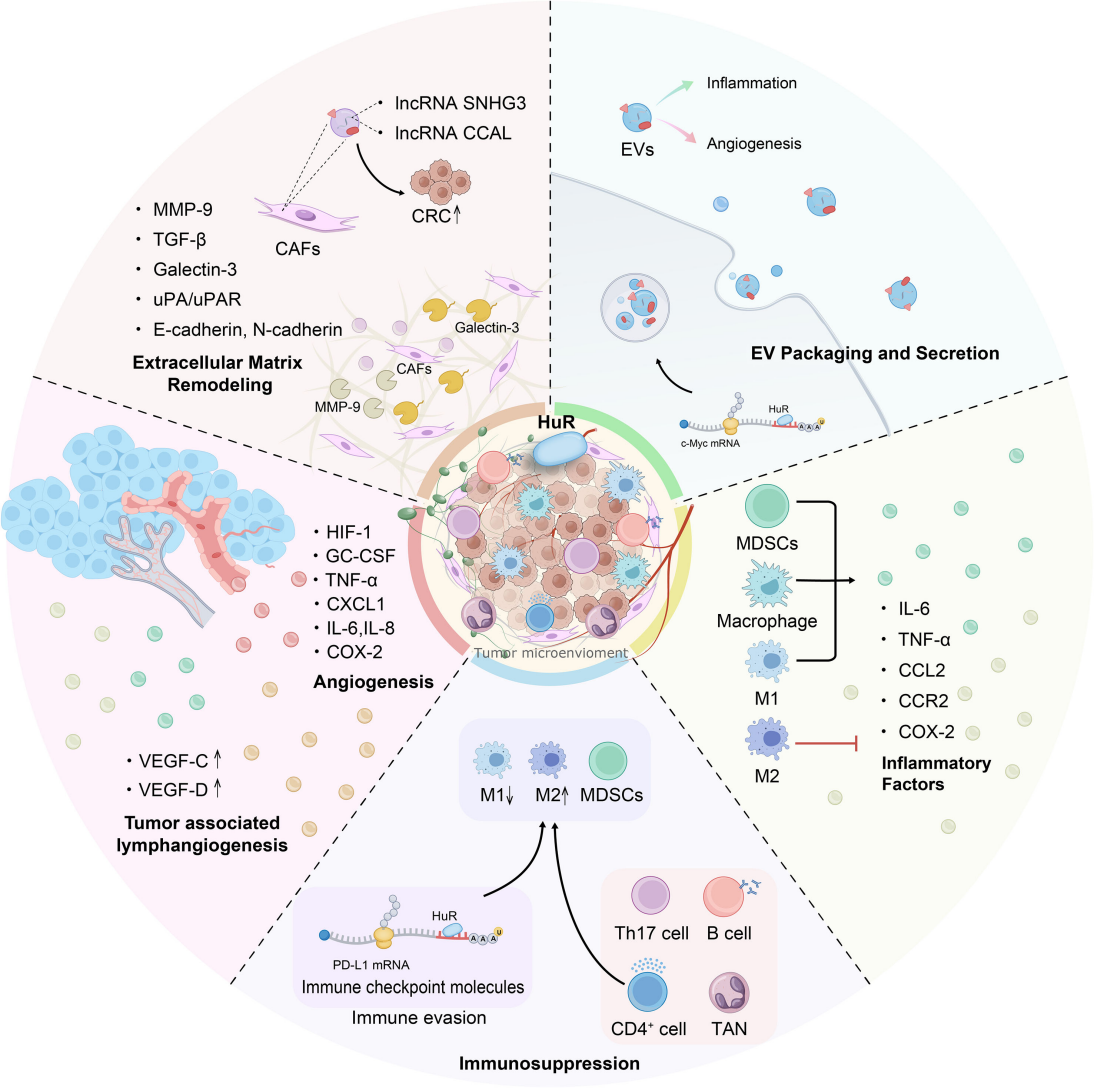
HuR exerts multifaceted regulatory effects within the CRC tumor microenvironment. This figure outlines the mechanisms by which HuR influences CRC progression from the perspective of the TME, as it promotes CRC development by enhancing inflammation, inducing immune suppression, remodeling the ECM, mediating EVs, and promoting angiogenesis and lymphangiogenesis. HuR interacts with immune cells such as Th17 and B cells, upregulating immune checkpoint molecules to induce immunosuppression, while it decreases M1 macrophages and promotes M2 polarization, simultaneously activating MDSCs and macrophages through direct and indirect mechanisms. Moreover, HuR enhances the secretion of inflammatory cytokines such as IL-6, IL-8, and TNF-α, thereby amplifying the inflammatory response in the TME. Furthermore, HuR promotes pro-angiogenic factors and VEGF expression, leading to vascular and lymphatic vessel formation. In the ECM, HuR facilitates CRC progression by upregulating galectin-3 and the uPA/uPAR pathway, increasing Snail and MMP-9 expression, and driving ECM remodeling, while CAFs in the ECM transfer lncRNAs via EVs to CRC cells to enhance tumor progression, and CRC-derived EVs further contribute to inflammation and angiogenesis. These five factors interact dynamically and collectively regulate CRC progression and metastasis.

HuR plays a critical role in CRC progression by regulating the TME. It influences inflammation, immune evasion, ECM remodeling, angiogenesis, and lymphangiogenesis, contributing to tumor growth, metastasis, and resistance to therapy (**Figure 4**). Although evidence shows that HuR regulates the expression of inflammatory factors such as IL-6 and TNF-α, the precise regulatory network and signaling cascades remain unclear. In addition to PD-L1, the involvement of HuR in immune evasion through other immune checkpoint inhibitors is not well understood. Moreover, the mechanisms by which HuR regulates immune cell secretion of cytokines and chemokines require further investigation. In terms of treatment, EVs have emerged as a promising therapeutic approach. However, additional research is needed to elucidate how HuR influences the biological composition of EVs and their role in modulating immune responses, inflammation, and angiogenesis within the tumor microenvironment.

## 6 HuR represents a potential therapeutic target for CRC

In the previous section, we detailed the molecular mechanism of HuR in CRC, revealing its central role and complex network in tumorigenesis and progression (169). Given the critical role of HuR in regulating the expression of tumor-related genes and the close association of its abnormal expression with the poor prognosis of patients, HuR has been regarded as a potential therapeutic target and a marker for therapeutic response and prognosis evaluation in CRC research (25). It has been reported that HuR is overexpressed in many tumor types, leading to poor prognosis of patients (170). Therefore, HuR inhibitors have become a hot spot in developing anticancer drugs (171). According to the current research, we can inhibit HuR mainly by the following strategies: inhibiting the expression of HuR, preventing the cytoplasmic translocation of HuR, interfering with the interaction between HuR and RNA, and affecting HuR dimerization (18, 172). The therapeutic strategies targeting HuR have been extensively reviewed by Jennifer M. Finan et al (26). This section will focus on the current research concerning HuR in CRC.

### 6.1 Inhibiting HuR cytoplasmic translocation

Previous studies have shown that HuR’s post-transcriptional regulatory function largely depends on its intracellular localization. Under normal conditions, HuR is primarily located in the cell nucleus. However, when cells are exposed to stress conditions such as hypoxia, inflammation, radiation, or other stimuli, HuR translocates to the cytoplasm, stabilizing target mRNAs and increasing their translation. Therefore, inhibiting HuR’s nucleocytoplasmic translocation is considered to specifically suppress its oncogenic role without affecting its physiological functions in the nucleus. MS-444 is a benzopyran derivative identified as a HuR inhibitor through high-throughput microbial, fungal, and plant extract screening. Initially found to be an inhibitor of myosin light chain kinase, MS-444 became one of the first HuR inhibitors characterized *in vitro* and *in vivo*. MS-444 interferes with HuR’s nuclear export signal (NES), preventing translocation from the nucleus to the cytoplasm. It also can bind to HuR’s first two tandem RNA RRMs (RRM1 and RRM2), successfully disrupting HuR’s binding with mRNA targets containing AREs and blocking HuR homodimerization, thereby preventing HuR from leaving the nucleus (25). HuR is retained in the nucleus and cannot stabilize mRNAs in the cytoplasm, reducing the expression of oncogenes such as COX-2, VEGF, and Cyclin D1 (173).

Pyrvinium pamoate is another compound capable of inhibiting HuR. Approved by the FDA for treating pinworm infections, Guo et al. found that dose-dependently inhibiting HuR accumulation in the cytoplasm causes HuR to remain in the nucleus. They further demonstrated that this compound reduces HuR phosphorylation levels by inhibiting the activity of checkpoint kinase 1 (Chk1) and cyclin-dependent kinase 1 (CDK1), thus blocking its translocation from the nucleus to the cytoplasm indirectly (174). Cryptotanshinone (CT), an active ingredient isolated from Salvia miltiorrhiza, has anti-inflammatory, antibacterial, antioxidant, and anti-platelet aggregation activities. Some studies have also shown that CT can inhibit the progression of a variety of tumors, including malignant melanoma, prostate cancer, hepatocellular carcinoma, and so on (175). Zhu et al. found for the first time that CT could interfere with the interaction between the NES of HuR and CRM1, preventing HuR from forming the HUR-CRM1 complex, thereby preventing its translocation from the nucleus to the cytoplasm, when studying the antiangiogenic effects of cryptotanshinone (166). In addition, many other compounds, such as compound SRI-42127 and Leptomycin B, have also been shown to inhibit HuR translocation, thereby achieving HuR inhibition (176, 177). However, these molecules have only been tested in mice or cell models and have not yet been tested in humans, so there is still a long way to go before their clinical application.

### 6.2 Inhibiting the HuR-RNA interaction

Various compounds and small molecules, such as CMLD-2, KH-3, and Dihydrotanshinone I (DHTS), have been identified as effective inhibitors of the HuR-RNA interaction, offering promising therapeutic avenues for reducing the stability of oncogenic mRNAs and inhibiting cancer progression (**Table 2**). CMLD-2 is one of the most effective inhibitors, identified through high-throughput screening (HTS) using fluorescence polarization (FP) assays of a compound library containing 6,000 compounds. It is a coumarin derivative that can directly bind to the RRM1 and RRM2 regions of the HuR protein, thereby disrupting the interaction between HuR and mRNAs such as Bcl-2, Msi1, and XIAP. This interaction disruption ultimately reduces the stability of these target mRNAs, leading to a decrease in their expression levels (180). KH-3 is a small molecule compound, N-(3-chloro-4-methoxyphenyl)-2-(3-pyridyl)pyridine-3-carboxamide, a novel HuR inhibitor obtained through structural optimization and chemical synthesis. It also decreased the expression of Snail mRNA and protein by destabilizing Snail mRNA, thus reducing the expression of key EMT transcription factors (such as Snail and Slug), inhibiting EMT, metastasis, and cancer stem cell (CSC) formation in pancreatic cancer cells (185).

**Table 2:** Inhibitors and mechanisms involved in the inhibition of HuR expression, cytoplasmic translocation, or its interaction with target mRNAs.

Action target	HuR inhibitors	Mechanism of action	Reference
Inhibition of HuR cytoplasmic translocation	MS-444	inhibitor through binding to HuR and impacting dimerization	(25)
	Pyrvinium pamoate (PP)	reduce the phosphorylation level of HuR	(174)
	Cryptotanshinone (CT)	interfere with the interaction between HuR’s NES and CRM1	(166, 175, 178)
	SRI-42127	inhibitor HuR dimerization	(176)
	Leptomycin B	highly specific binding and inhibition of CRM1	(172, 179)
Inhibition of HuR–RNA interaction	CMLD-2	disrupt HuR interaction with Bcl-2, Msi1, and XIAP mRNA	(180)
	KH-3	interact with the RRM1 and RRM2 domains of HuR,	(181)
	dihydrotanshinone-I (DHTS)	limits the association rate of HuR with RNA	(182)
	quercetin	inhibit HuR: ARE (TNF-α) complex formation	(134)
	suramin	The specific mechanism is unknown	(110)
inhibition of HuR expression	miR-519	bind to specific sites on the 3’ UTR of HuR and induce the formation of the RNA-induced silencing complex (RISC)	(66, 73)
	miR-22	Impede the assembly of the translation initiation complex and inhibit the translation efficiency of HuR protein	(64)
	miR-125a	target the 3’ UTR of HuR, regulate the ubiquitin-proteasome pathway, and increase the degradation rate of HuR mRNA.	(183)
	miR-16	Possibly suppresses the expression of c-Myc and NF-κB, indirectly reducing the transcription level of HuR	(5, 48, 76)
	miR-34a	target the 3’ UTR of HuR, regulate the ubiquitin-proteasome pathway, and increase the degradation rate of HuR mRNA.	(87, 184)

HuR, Human Antigen R; NES, Nuclear Export Signal; CRM1, Chromosome Region Maintenance 1 (Exportin-1); RRM1, RNA Recognition Motif 1; RRM2, RNA Recognition Motif 2; Bcl-2, B-cell lymphoma 2; XIAP, X-linked inhibitor of apoptosis protein; TNF-α, Tumor Necrosis Factor alpha; RISC, RNA-induced silencing complex; UTR, Untranslated Region; c-Myc, cellular Myelocytomatosis oncogene; NF-κB, Nuclear Factor Kappa B; Msi1, Musashi-1; PP, Pyrvinium pamoate; CT, Cryptotanshinone; DHTS, dihydrotanshinone-I.

Dihydrotanshinone I (DHTS), a natural product derived from Salvia miltiorrhiza, is another inhibitor of the HuR-RNA interaction. It directly binds to the RNA-binding domain of HuR, a conformational change in HuR, keeping it in a “closed” state unfavorable for RNA binding, thereby hindering HuR’s function. By reducing HuR’s stabilization of mRNAs for genes such as COX-2 and Cyclin D1, DHTS inhibits tumor cell proliferation and the inflammatory response. *In vivo* experiments showed that DHTS significantly inhibited tumor growth and reduced tumor volume in CRC xenograft models. In addition to demonstrating anticancer activity in CRC, DHTS also exhibited cytotoxicity in breast, pancreatic, and glioma cancers (182). Researchers have also found that bioactive flavonoids such as quercetin can disrupt the binding between HuR and inflammatory cytokine mRNAs, reducing inflammation and tumor invasiveness (134). Suramin, on the other hand, exerts its anticancer effects in oral cancer HSC-3 cells by competitively disrupting the binding between HuR and ARE-containing mRNAs (such as those encoding cyclin A2 and cyclin B1). The impairment of HuR-mediated mRNA stability ultimately attenuates the malignant phenotype, as demonstrated by markedly decreased motile and invasive activities in suramin-treated tongue carcinoma cells (110). RNA aptamers have been demonstrated to bind the Armadillo repeat domain of β-catenin with high specificity and affinity in CRC. By competitively inhibiting its interaction with natural cellular RNA partners (e.g., sequences in the COX-2 3’-UTR) and disrupting the formation of complexes with HuR, they effectively impair its oncogenic functions. This mechanism highlights the significant therapeutic potential of designing RNA aptamers to inhibit HuR in an analogous manner (50, 186).

### 6.3 MicroRNAs and LncRNAs as targets to modulate HuR

MicroRNAs (miRNAs) play an important role in post-transcriptional gene regulation, and a variety of miRNAs have been found to regulate HuR expression. In 2008, Abdelmohsen K et al. first found that miR-519 could interact with the coding region (CR) and 3 ‘UTR of HuR mRNA in HCT116 and RKO (CRC), HeLa (cervical cancer), and A2780 (ovarian cancer) cells (73). By inhibiting the expression and translation of HuR, it indirectly reduces the expression of HuR target mRNAs, such as cyclin, growth factors, and mitogenic transcription factors. Subsequently, miR-22 has also been found to directly inhibit the translation of HuR and reduce the expression of HuR protein, thereby inhibiting the proliferation and migration of CRC cells (64). Other miRNAs, such as miR-31, miR-145, miR-155, miR-125, and miR-34a, can exert regulatory effects by antagonizing the stabilizing effect of HuR on target mRNAs (187).

Beyond miRNAs, lncRNAs can also influence the functional regulation of HuR in CRC (188). OCC-1 can disrupt the stability of HuR, thereby inhibiting CRC growth. It also enhances the interaction between the E3 ubiquitin ligase β-TrCP1 and HuR, making HuR more susceptible to ubiquitination and degradation (36), which in turn reduces the levels of HuR and its target mRNAs, including those directly associated with cancer cell growth. Similarly, lncRNA GMDS-AS1 stabilizes STAT3 mRNA by preventing its ubiquitination degradation through binding to HuR (93). The stabilization of HuR not only activates the STAT3/Wnt signaling pathway to promote CRC cell survival and proliferation and suggests a potential link between CRC and chronic inflammation through lncRNAs R. Furthermore, lncRNA SPRY4-IT1 can enhance HuR’s interaction with mRNA related to tight junction (TJ) proteins, promoting the expression of claudin-1, claudin-3, occludin, and JAM-1, thus maintaining intestinal epithelial barrier function and inhibiting tumorigenesis and progression (94).

### 6.4 Gene interference technology

Gene interference technology is another approach to reduce HuR expression in cancer cells. Gene suppression of HuR using knockdown (e.g., shRNA, siRNA) and knockout (e.g., CRISPR/Cas9) methods has been shown to inhibit tumor growth both *in vitro* and *in vivo (* 189). Danilin et al. found that siRNA targeting HuR suppressed HuR and its targets (including Bcl-2) expression, thereby inhibiting CRC cell survival and promoting apoptosis. In breast cancer, the knockdown of HuR inhibited cell invasion and reduced lung metastasis (190). Similar results have been found in pancreatic cancer, where low HuR levels inhibit tumor growth and invasion (191).

CRISPR/Cas9-mediated HuR knockout is a new method for studying HuR biology, effectively avoiding issues such as the instability of delivery vectors in shRNA-induced knockdown or siRNA transient transfections (192). Shruti Lal et al. used CRISPR/Cas9 technology to delete HuR from pancreatic ductal adenocarcinoma (PDA) and CRC cells (193). This system effectively targets specific genomic sequences and generates disruptive double-strand breaks (DSBs). Results showed that, compared to wild-type and CRISPR control cells, HuR protein expression was reduced. ASOs are short synthetic nucleic acid fragments, usually composed of 15–25 nucleotides. ASOs interfere with specific mRNA or miRNA sequences by complementary binding, forming a hybrid double-strand to disrupt the target RNA’s function (194). ASO technology is a powerful tool for gene expression regulation. By designing ASOs complementary to HuR mRNA, its translation can be inhibited, and HuR protein expression can be reduced. This technology has been validated in microglia-mediated spinal cord neuroinflammation, where it promotes anti-inflammatory and neuroprotective responses (195). It may also have the potential for use in CRC treatment in the future.

### 6.5 Combination therapy

With more profound research into HuR, increasing evidence suggests that HuR also plays a critical role in CRC’s resistance to chemotherapy and radiotherapy. However, chemotherapy alone may not be sufficient to overcome drug resistance. Therefore, combination therapy strategies have become necessary. By targeting HuR and combining it with chemotherapy drugs, treatment efficacy may be improved, resistance overcome, and patient survival rates increased. One combination therapy strategy is to use HuR inhibitors in conjunction with conventional chemotherapy drugs (196). Wu et al. found that combining CMLD-2 with 5-fluorouracil (5-FU) significantly inhibited the proliferation of CRC cells and enhanced their sensitivity to 5-FU (197). This is because CMLD-2 inhibits the binding of HuR to the ABCB1 gene mRNA, leading to reduced expression of ABCB1, which encodes P-glycoprotein (P-gp). ABCB1 plays a crucial role in tumor resistance, and its downregulation helps overcome resistance (198). Similarly, PP, when combined with chemotherapy drugs (such as cisplatin, doxorubicin, vincristine, and oxaliplatin), can enhance chemotherapy-induced DNA double-strand breaks, thereby increasing cytotoxicity in bladder cancer cells and synergistically inhibiting the growth of bladder tumor xenografts in mouse models (174). *In vitro* experiments showed that the small molecule HuR inhibitor MS-444 could inhibit HuR homodimerization and cytoplasmic translocation, affecting HuR-mediated overexpression of PIM1. This, in turn, enhanced the sensitivity of pancreatic ductal adenocarcinoma (PDA) cells to oxaliplatin and 5-FU under physiological hypoxic conditions (191).

Another combination strategy involves nanoparticle-based delivery systems to deliver HuR siRNA with chemotherapy drugs. Liposomes are among the earliest-developed and most mature delivery vectors due to their excellent biocompatibility and low immunogenicity. Lipid nanoparticles can couple with siRNA to prevent degradation and facilitate the targeted siRNA delivery, effectively silencing the target mRNA. As cancer cells commonly overexpress folate receptors, researchers have designed folate-FA ligand-conjugated delivery systems by modifying the surface of liposomes with tumor-specific folate ligands. This approach enhances the uptake of siRNA, drug molecules, and gold nanoparticles by cancer cells, achieving targeted delivery. Additionally, labeling folate receptors aids in the early screening of CRC liver metastasis patients. Danilin et al. found that HuR siRNA decreased the expression of galectin-3, β-catenin, cyclin D1, Bcl-2, P-gp, MRP1, and MRP2 in CRC cells treated with doxorubicin. siRNA-mediated HuR silencing enhanced the accumulation of doxorubicin in CRC cells (199). On the other hand, HuR overexpression negated this effect. Furthermore, siHuR significantly enhanced doxorubicin-induced apoptosis by increasing reactive oxygen species (ROS), boosting its cytotoxic effects. Badawi et al. also found that siHuR-mediated HuR downregulation significantly increased the sensitivity of CRC cells to paclitaxel (29).

Most studies targeting HuR report enhanced therapeutic efficacy and increased apoptosis, though a few fail to reproduce these effects—likely reflecting intrinsic resistance. Integrating current evidence, we infer that such resistance arises from compensatory activation of parallel RBPs or signaling cascades, notably AUF1 (98), TIAR (101), PI3K/Akt (87, 92), and MAPK/ERK pathways, which may sustain cell survival after HuR inhibition. Overexpression of drug efflux pumps such as P-gp may further limit intracellular drug exposure, reducing the effectiveness of HuR inhibitors (198). HuR expression also varies across CRC subtypes, being reduced in MSI-H and elevated in MSS tumors, consistent with their divergent genomic stability and post-transcriptional regulation. In MSS CRC, HuR likely promotes chemoresistance by stabilizing anti-apoptotic and efflux-related transcripts (200). Collectively, these findings position HuR as a context-dependent therapeutic target rather than a universal one. HuR inhibition is most effective when chemoresistance relies on anti-apoptotic escape or ABC transporter–mediated efflux (e.g., anthracyclines, platinums, topoisomerase inhibitors). Tumors with cytoplasmic HuR enrichment and HuR-dependent activation of survival or efflux programs are especially susceptible to small molecules that block HuR–RNA binding or cytoplasmic translocation, thereby restoring chemosensitivity (18).

Additionally, combining radiotherapy with HuR RNA interference technology is a potential strategy. Radiotherapy is an essential method for the treatment of CRC, but some tumor cells that are resistant to radiotherapy may survive and become the source of tumor recurrence. Therefore, exploring the mechanism of radiotherapy tolerance and finding strategies to improve the radiosensitivity has become the focus of research (201). Recent studies on the relationship between HuR and radiotherapy have shown that the expression of caspase-2 in CRC cells significantly increased after silencing HuR, and CRC cell lines DLD-1 and HCT-15 are more sensitive to radiation-induced apoptosis (29). In addition, the reduction of HuR significantly increased the number of γH2AX/53BP1 positive foci induced by radiation, suggesting an increase in DNA damage (29, 202). The efficacy of radiotherapy combined with targeted therapy has also been verified in triple-negative breast cancer. These *in vitro* studies on solid tumors showed that siRNA-mediated HuR knockdown increased the sensitivity of CRC cells to radiotherapy (201).

**Figure 5:**
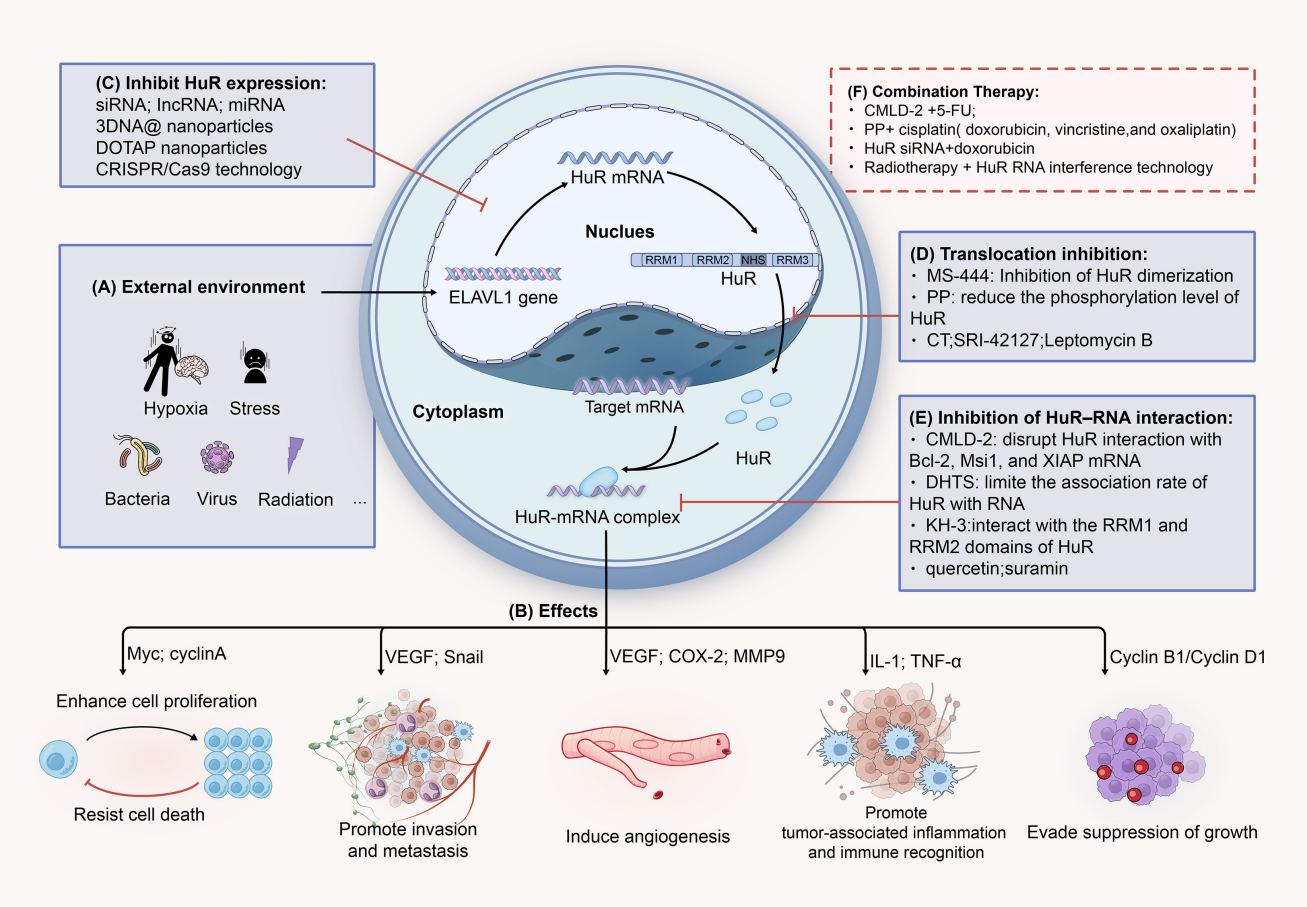
HuR represents a potential therapeutic target for CRC. **(A)** Hypoxia, inflammation, radiation, and other stress conditions can promote the expression of the ELAVL1 gene, further facilitating the translocation of HuR to the cytoplasm. In the cytoplasm, HuR stabilizes target mRNAs, thereby increasing their translation. **(B)** HuR-mRNA promotes tumor cell growth and proliferation, invasion and metastasis, immune evasion, angiogenesis, and other processes through various signaling pathways and molecular mediators. **(C)** SimRNA, IncRNA, miRNA, and others can suppress the expression of HuR by interfering with the stability of HuR RNA or by binding to its specific structural regions. **(D)** MS-444, PP, CT, and others can block the cytoplasmic translocation process of HuR by interfering with its nuclear export signal (NES). **(E)** CMLD-2, DHTS, KH-3, and others can bind to the RRM1 and RRM2 regions of the HuR protein, disrupting its interaction with target mRNAs, leading to a decrease in their expression levels. **(F)** Combining HuR inhibitors with conventional chemotherapy drugs or radiotherapy (such as CMLD-2 + 5-FU) can enhance treatment efficacy and overcome resistance.

Totally, HuR inhibitors have demonstrated significant potential in CRC therapeutic research. Strategies such as suppressing HuR gene expression, disrupting HuR-target mRNA interactions, employing gene interference technologies, and combining HuR inhibition with radiotherapy/chemotherapy have shown efficacy in inhibiting tumor cell proliferation, metastasis, and drug resistance. We have illustrated these mechanisms and processes in **Figure 5**. Despite these promising findings, the clinical translation of HuR inhibitors faces critical challenges. First, as HuR is the only ubiquitously expressed member of the ELAVL family across human tissues and participates in diverse biological processes, from embryogenesis to cell death, its essential roles in normal cellular functions, particularly in the digestive and immune systems, raise concerns about off-target effects. Non-specific inhibition may damage healthy cells, exacerbate treatment toxicity, and compromise patient quality of life and therapeutic tolerance. Thus, designing highly selective HuR inhibitors with minimal toxicity remains a pressing challenge. Second, HuR enhances cancer cell survival by upregulating anti-apoptotic genes (e.g., BCL-2, MCL-1) and modulates chemoradiotherapy resistance through interactions with miRNAs and their target genes. Overcoming such resistance by leveraging HuR as an adjuvant therapeutic target could offer novel strategies for CRC management. Third, HuR expression levels and functional roles may vary significantly across CRC subtypes, potentially leading to divergent mechanisms of action in distinct tumor contexts. Elucidating these subtype-specific molecular mechanisms through detailed mechanistic studies is imperative. Finally, the long-term efficacy of HuR inhibitors, including sustained tumor suppression and prevention of recurrence, remains unverified. CRC treatment requires a holistic approach addressing drug resistance, tumor microenvironment dynamics, and other factors. Consequently, clinical evaluation of HuR inhibitors must prioritize not only therapeutic efficacy but also tolerability and durable outcomes.

## 7 HuR serves as a CRC diagnostic biomarker

Elevated levels or altered localization of HuR have been observed in various types of cancers, including CRC, making it a promising candidate for CRC diagnosis and potentially for prognosis. The cytoplasmic status of HuR in tumor cells has also been shown to correlate with prognosis in many tumor types. For example, Denkert et al. found that cytoplasmic HuR was positively correlated with COX-2 expression, lymphatic invasion, lymph node metastasis, and tumor grade in CRC, which suggests a poorer prognosis (133). Furthermore, a study has found that cytoplasmic HuR levels correlate with Gleason score, T stage, and metastasis in hormone-naive prostate cancer tissues, and it is a potential predictor of biochemical recurrence after radical prostatectomy (203). These studies all support the view that cytoplasmic HuR promotes tumor progression and recurrence and is associated with poor patient survival, disease-free survival, and metastasis-free survival. Therefore, it can be used as an independent diagnostic biomarker to assess malignancy and prognosis.

Based on numerous previous studies, HuR can also serve as a biomarker to predict CRC response to chemotherapy and radiotherapy. In CRC, To et al. found that most patients who were unresponsive to 5-FU chemotherapy had higher levels of ABCG2 and HuR expression and lower expression of miR-519c in their tumor samples. High HuR levels promote tumor cell resistance to doxorubicin (66). They also found that cytoplasmic HuR stabilizes the mRNA of cell cycle genes like Cyclin D1, promotes cell proliferation, and reduces doxorubicin’s efficacy (105). A study on pancreatic cancer patients who underwent potentially curative pancreatic resection also showed that cytoplasmic HuR staining is a positive predictor of gemcitabine sensitivity and good prognosis (185). Given its potential value in diagnosis, prognosis, and treatment response prediction, HuR is expected to become an important molecular biomarker for CRC (204). However, further clinical studies are needed to verify the feasibility and effectiveness of its clinical application.

## 8 Challenges and future directions

HuR is known to play a crucial role in the initiation and progression of CRC, emerging as a potential therapeutic target in cancer treatment. Over the past decade, research utilizing small molecules or siRNA to inhibit HuR has advanced significantly. However, despite substantial progress in lung (27), ovarian (205), breast, prostate (206), and other cancer types, the application of HuR inhibition in CRC remains relatively underexplored. To date, no HuR inhibitors have been approved by the FDA, nor have any HuR-based therapy entered clinical trials. Consequently, no effective treatments targeting HuR are available. Many HuR inhibitors have undergone proof-of-concept validation in various animal tumor models and other disease models, but further preclinical studies are required to optimize these models. In translating these findings to clinical settings, additional exploration of strategies and potential challenges is necessary.

HuR is the only member of the ELAVL family that is ubiquitously expressed in all human tissues, which poses a challenge in minimizing off-target effects during treatment. To avoid damage to healthy cells and reduce the risk of increased toxicity, one approach is to design highly specific small-molecule inhibitors or peptide molecules targeting cancer-specific HuR-binding sites on mRNAs. For example, inhibitors targeting tumor-specific phosphorylation sites of HuR (e.g., Ser202 (135) or Thr118 (30)) could selectively affect cancer cells without interfering with normal cell mRNA targets. Additionally, ASOs, siRNAs, or miRNAs can be engineered with tumor-specific promoters or microenvironment-responsive elements, significantly reducing non-specific distribution while increasing drug concentrations at the tumor site, thus optimizing bioavailability and therapeutic efficacy with minimal cytotoxicity. To further enhance therapeutic specificity and overcome drug delivery barriers, nanotechnology has gained considerable attention in cancer treatment. Nanoparticle systems encapsulating HuR inhibitors, siRNA, or ASOs could improve the stability and bioavailability of these agents *in vivo* for CRC treatment. Additionally, leveraging the unique characteristics of the tumor microenvironment, such as the design of pH-sensitive nanoparticles (e.g., chitosan (207)) that release drugs in acidic conditions or under specific enzymatic activities, could improve targeted drug delivery. Exosomes, with their excellent biocompatibility and low immunogenicity, could also serve as natural nanoparticles for delivering HuR-targeted siRNAs or miRNAs.

Finally, the challenge of drug resistance is inevitable in cancer therapies, though there has been little research on this issue concerning HuR-targeted treatments. Drawing on the biological properties and mechanisms of HuR, along with insights into resistance in other targeted therapies, we propose several strategies to mitigate resistance to HuR-targeted therapy: (1) combining HuR inhibitors with inhibitors of other signaling pathways to block compensatory pathway activation, thus reducing the potential for tumor cells to regain survival capacity through alternative routes; (2) using computational simulations to design blockers targeting HuR’s RRM or HNS domains based on its crystal structure, preventing mRNA binding, which presents a promising avenue for further research; (3) employing inhibitors of efflux pumps, such as P-gp, to increase the intracellular concentration of HuR inhibitors or modulating autophagy pathways to enhance their therapeutic effects and overcome resistance.

## 9 Conclusion

In this review, we first explore the pivotal role of the RNA-binding protein HuR in the initiation and progression of CRC from three key perspectives: mRNA stability and translation, cell proliferation and survival, and the tumor microenvironment. As a potential therapeutic target, HuR offers promising opportunities for the treatment of CRC. However, to fully exploit its therapeutic potential, further research is needed to gain a deeper understanding of its complex regulatory mechanisms, identify specific molecular targets, and overcome the challenges involved in translating these findings into clinical practice.
